# Traditional Chinese medicine for post-stroke depression: A systematic review and network meta-analysis (Protocol)

**DOI:** 10.1097/MD.0000000000013840

**Published:** 2018-12-28

**Authors:** Wanlin Huang, Xiaoqin Liao, Jinhui Tian, Jing Wu, Yawei Shan, Weini Zhou

**Affiliations:** a Shanghai University of Traditional Chinese Medicine, Shanghai; b Lanzhou University, Centre of Evidence Based Medicine, Lanzhou City, Gansu, China.

**Keywords:** network meta-analysis, post-stroke depression, traditional Chinese medicine

## Abstract

**Background:**

Post-stroke depression (PSD) is common in stroke survivors, with significantly negative effects and serious impairments in terms of personal and social functioning. While both pharmacological and traditional Chinese medicine (TCM) interventions have been administered for PSD, there is still uncertainty about the balance between these and what treatment strategy should be preferred in clinical practice. Therefore, we aim to compare and rank, describing the protocol of a systematic review and network meta-analysis (NMA), the commonly used TCM interventions for PSD.

**Methods and analysis:**

We will search CENTRAL (the Cochrane Central Register of Controlled Trials), CINAHL, Embase, PubMed, CBM and PsycINFO, the US National Institutes of Health and the World Health Organisation International Trials Registry Platform search portal from inception to November 2018. There will be no restrictions on language, publication year or publication type. Only randomized clinical trials (RCTs) accessing any TCM treatments against active comparator or other controls for PSD will be included. The primary outcomes will be efficacy (the total number of participants, declining more than 50% on the total score between baseline) and acceptability of treatment (dropout rate due to any cause). A Bayesian NMA will be performed to compare all relative outcome of different TCM interventions. we will conduct the network meta-regression meta-analyses of data on the sex ratio, the types of stroke and the treatment duration of TCM interventions. Potential explanations in extra subgroup analyses according to the results of heterogeneity and inconsistency will be explored, and sensitivity analyses will be conducted to assess the robustness of the findings.

**Trials registration number:**

PROSPERO CRD42018082400.

**Conclusion:**

Our study will generate evidence for TCM in the treatment of PSD and help to reduce the uncertainty about the effectiveness of PSD management, which will encourage further suggestions for TCM clinical practice or guideline.

## Abstract 2

Supplemental Digital Content is available in the text

## 1 Introduction

Depression is one of the most common mood disturbance after stroke, with up to 39% prevalence rate reported in previous local studies.^[1,2]^ Pooled data from 61 studies including more than 25,000 subjects with a clinical diagnosis of stroke suggest that approximately 31% of stroke survivors experience depression within the 5 years following stroke.^[3]^ Leading contributors to total years lost to disability based on the global burden of diseases report, such comorbidity of stroke with depression has been described as the ‘double burden’ of stroke.^[4]^ The negative impact of post-stroke depression (PSD) on quality of life, characterized by a range of cognitive and behavioral symptoms, as well as increased mortality, highlight the need for effective treatment.^[5–7]^ So it is highly concerning that up to 60% of patients do not respond adequately to pharmacological antidepressant treatment ^[4]^ and 30% do not adhere to medication.^[5]^ Patients have expressed the view that there is an over-reliance on prescribed antidepressant medication and they are keen to have a range of possible treatment choices.^[6]^ The net result is that an interest in non-pharmacological options is growing.

Despite the pathogenesis of PSD is well documented, no study has interpreted the pathogenesis from a single systematic aspect, because the development of PSD involves multiple systems.^[8]^ The therapeutics of PSD is still unclear and the routine use of prophylactic antidepressants is not recommended as optimal timing and duration of interventions remain to be illuminated and the risk-benefit has not been clearly established.^[9]^ In addition, it deserves attention greatly that up to 60% of patients do not respond adequately to antidepressant treatment^[10]^ and 30% do not adhere to drug therapy.^[11]^ The net result is that an interest in complementary therapies is increasing.^[12]^ People with PSD may consider using Traditional Chinese Medicine (TCM) treatments, such as acupuncture, ear-acupoint application therapy, and moxibustion, and an increasing body of research has been undertaken to assess the effectiveness of TCM therapies for treatment of individuals with PSD.

A British trial of acupuncture or counseling for depression provides evidence based on 755 patients, the largest trial to date.^[13]^ The results demonstrated that standard care plus acupuncture, when compared with usual care, is associated with statistically significant short- to medium-term benefits with little risk of harm. The more recent Cochrane review on acupuncture for depression provided an update on the evidence in 2018.^[14]^ This updated review found the reduction in severity of depression was less when acupuncture was compared with sham acupuncture control than when acupuncture was compared with no treatment control.

In studies from the Chinese team on depression,^[15,16]^ the mechanism of auricular vagus nerve stimulation (VNS) was found to be analogous to cervical VNS in terms of pathways. Another study successfully established the animal model of chronic-stress-induced depression and observed from an animal behavior perspective that acupuncture at the vagus nerve distribution area could significantly improve symptoms of rats with depression, which represented a substantial advance at the psychological level.^[17]^

Moxibustion is one of the most representative nonpharmacologic therapies, particularly in traditional medicine in East Asia, including China, Japan, and South Korea.^[18]^ China has provided huge amounts of data in this field, and according to a research by Woo et al,^[19]^ acupuncture plus moxibustion is frequently referred therapeutic modalities for the treatment of depression. Still, other studies have found that moxibustion plus acupuncture therapy is superior to sham acupuncture^[20]^ or to a wait list control.^[21]^

Despite numerous TCM interventions evaluated in previous randomized controlled trials (RCTs) to treat PSD, the majority has not been quantitative analyzed in head-to-head comparisons. Thus, we employed a network meta analysis (NMA) of all RCTs of TCM treatment approaches for PSD, including ear-acupoint application therapy, moxibustion, etc, to synthesize all this evidence and perform an integrated rank of available TCM treatments for PSD.

## 2 Methods

This systematic review protocol will be prepared to underlie the Preferred Reporting Items for Systematic Review and Meta-Analysis Protocols (PRISMA-P) guidance.^[22,23]^ This protocol will be in accordance with the recommendations of the PRISMA Extension Statement for Reporting of Systematic Reviews Incorporating Network Meta-analyses of Health Care Interventions.^[24]^ The NMA protocol has been registered with the International Prospective Register of Systematic Reviews in January 2018: CRD42018082400.

### 2.1 Criteria for included studies

#### 2.1.1 Types of studies

We will only involve RCTs and quasi-RCTs, including the first phase of cross-over trials as well as cluster-randomized trials. As it is difficult to use a double-blind design for patients in trials of TCM therapy alone or the combination of antidepressant and TCM therapy, only trials in which raters were blinded or subjects were assessed by self-rating depression scales (SDS) will be included. Studies should be available in full papers and peer-reviewed.

#### 2.1.2 Types of participants

Individuals with a recent or past history of ischemic or hemorrhagic stroke, of both sexes with a diagnosis of depressive disorder, based on standardized diagnostic criteria (eg, the Diagnostic and Statistical Manual of Mental Disorders or the International Classification of Diseases) will be eligible for recruitment.^[25–29]^ Enrolled participants will be aged over 18 years, medically stable, able to give informed consent and follow multiple-staged commands. Studies will be excluded, where depressive disorder was not formally diagnosed. RCTs recruiting subjects with an overall sample size of fewer than 10 participants will also be excluded.

#### 2.1.3 Types of interventions

For TCM interventions, we will include 18 technologies of TCM according to the nursing guide formulated by State Administration of TCM of China, for example, scraping, cupping, moxibustion, sticking acupuncture points, Chinese medicine iontophoresis, massage, ear-acupoint application therapy, and acupuncture point injection. Table 1 provides the detailed description of TCM interventions.

**Table 1:**
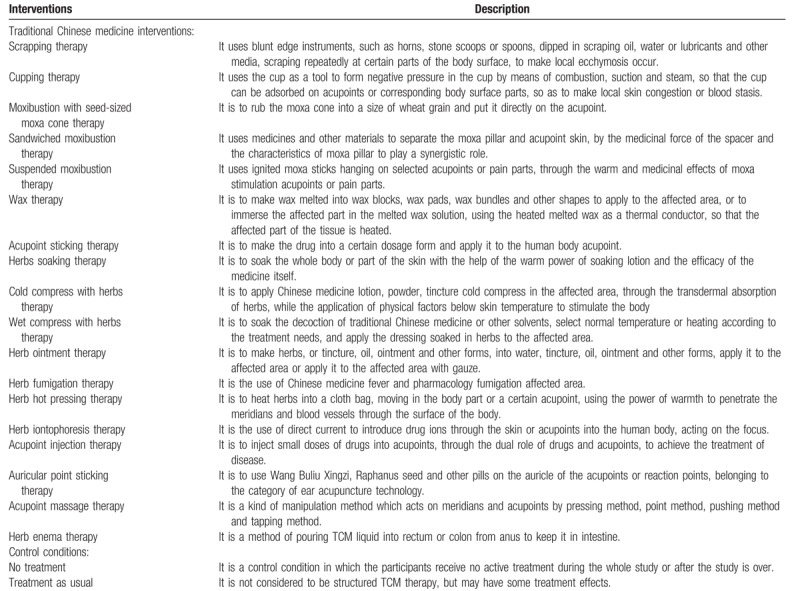
The detailed description of traditional Chinese medicine interventions and control conditions.

All RCTs comparing any active intervention (TCM interventions or their combinations) with either active comparators or control conditions for treatment of PSD will be included. Trials comparing the same type of TCM interventions, but at different numbers of therapeutic sessions, different acupuncture points (similar but not identical), and different treatment conditions (with or without nurses’ involvement) will be considered as the same node in the network analysis. We are working on the assumption that any recruiting participants, in principal, is under the same condition to be randomized to any of the interventions in the synthesis comparator set.

**Figure 1:**
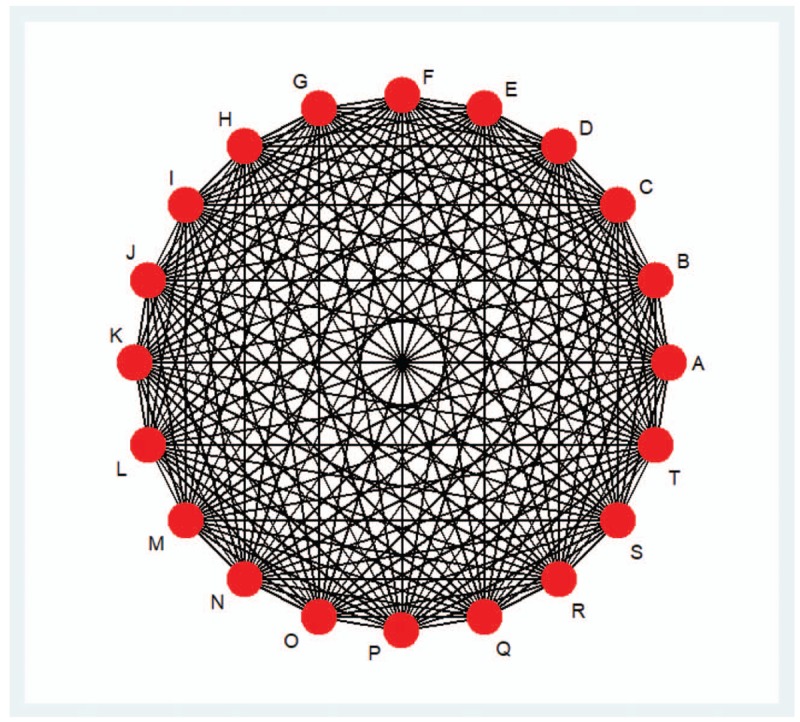
Possible interventions eligible for the ideal network plot. 1. The filled circles represent different interventions. A: Scrapping therapy; B: Cupping therapy; C: Moxibustion with seed-sized moxa cone therapy; D: Sandwiched moxibustion therapy; E: Suspended moxibustion therapy; F: Wax therapy; G: Acupoint sticking therapy; H: Herbs soaking therapy; I: Cold compress with herbs therapy; J: Wet compress with herbs therapy; K: Herb ointment therapy; L: Herb fumigation therapy; M: Herb hot pressing therapy; N: Herb iontophoresis therapy; O: Acupoint injection therapy; P: Auricular point sticking therapy; Q: Acupoint massage therapy; R: Herb enema therapy; S: No treatment; T: Treatment as usual. 2. The lines between the circles represent that there exist some researches in the study of the difference between the intervention effect of different methods of intervention.

An ideal network plot, with all expected interventions, has been generated to simulate a fully connected network (Fig. 1).

#### 2.1.4 Types of outcome measures

##### 2.1.4.1 Primary outcomes

Efficacy (as dichotomous outcome), measured by the total number of participants, achieving the criteria of remission that is defined as declining more than 50% on the total score between baseline on a standardized observer-rating scale for depression.^[30]^

Acceptability, defined as the proportion of recruited patients who withdrew from the study due to any reason during the delivery of the intervention.

##### 2.1.4.2 Secondary outcomes

Efficacy (as a continuous outcome), measured by the overall mean change scores on depressive symptom scales (self-rated or assessor-rated), such as SDS^[31]^ from baseline to endpoint.

Tolerability, defined as the proportion of recruited patients who quitted from treatment by any adverse events during the delivery of the intervention.

Activities of daily living (as a continuous outcome), measured by Barthel index^[32]^ or any ADL measured using established and validated assessment tools.

### 2.2 Search strategy and study selection

We will search for all published and unpublished RCTs, without language or date restriction. Published RCTs will be searched in the following electronic databases: CENTRAL, CINAHL, Embase, PubMed, CBM and PsycINFO. The electronic search will be supplemented with manual searches for published, unpublished and ongoing RCTs in the following electronic sources of trials: the US National Institutes of Health (www.clinicaltrials.gov), The World Health Organisation International Trials Registry Platform search portal (www.who.int/trialsearch/Default.aspx) and Google Scholar. We will hand search reference lists of relevant trials and systematic reviews, as well as journals and conference abstracts, retrieved by the search and contact experts in the field to obtain additional data. A draft PubMed search strategy is included in Appendix 1. After the PubMed strategy is finalized, it will be adapted to the syntax and subject headings of the other databases.

Two reviewers (HWL and ZWN) will independently review titles and abstracts retrieved by the search. The trial will be excluded If both reviewers agree that it does not meet eligibility criteria. We will obtain the full text of all remaining articles and use the same inclusion criteria to determine whether, if any, to exclude in the process. Any disagreements will be resolved via discussion with a third member (LXQ). In the characteristics of excluded studies list, the reasons for exclusion of trials will be clearly explained.

### 2.3 Data extraction

For all included trials, two independent reviewers (HWL and ZWN) will extract data using a data extraction form and summarise trial characteristics in tables, including study characteristics (eg, first listed author, publication year, title, publication type, publication journal, country), patient characteristics (eg, diagnostic criteria, comorbidities, the age of patients, patient setting, the number of patients, the gender of patients), intervention details and the outcomes. Any disagreements will be resolved via discussion with a third review author (LXQ). The authors of the studies will be contacted for further information, as required.

### 2.4 Risk of bias assessment

Two authors (HWL and ZWN) will independently assess risk of bias for each selected study in accordance with the Cochrane ‘Risk of bias’ assessment tool^[33]^ which includes six domains: selection (random sequence generation and allocation concealment); performance (blinding of participants and personnel); detection (blinding of outcome assessors); attrition (incomplete outcome data); reporting (selective reporting); and other bias. Disagreements will be resolved by a third investigator (LXQ). The conclusions will be presented in the ‘Risk of bias’ table, which will be incorporated into the interpretations of results by means of sensitivity analyses.

### 2.5 Statistical analysis of study data

We will perform pairwise meta-analyses of direct evidence using the random-effects model with Stata V.14.0, while a random-effects NMA within a Bayesian framework will be performed, using Markov chain Monte Carlo in WinBUGS V.1.4.3. Where different measures are used to assess the same outcome, dichotomous outcomes data will be analyzed by calculating Mantel–Haenszel odds ratios (ORs) and continuous outcomes will be pooled with standardized mean difference (SMD). We will present 95% confidence intervals for all outcomes.

The data will be managed on an intention-to-treat (ITT) basis as far as possible. Attempts will be made to obtain missing data from the trial authors. Where data are unobtainable, we will assume that an event, without a reported outcome, has not occurred in participants and we will analyze only the available data.

Furthermore, we will calculate the ranking probabilities for all treatments of being at each possible rank for each intervention, using the surface under the cumulative ranking (SUCRA), where the SUCRA values can range from zero to one.

### 2.6 Measures for heterogeneity

The presence of clinical and methodological heterogeneity will be validated by applying with the method of descriptive statistics for study population characteristics. As to transitivity assumption in network meta-analysis (NMA), we will consider whether the interventions and characteristics across all eligible trials are sufficiently similar to each other. Additionally, statistical heterogeneity within each pairwise comparison will be assessed using the I^2^ statistic and only an I^2^ above 50% would be taken to indicate a problem with substantial heterogeneity.^[33,34]^

### 2.7 Measures for inconsistency

Another key assumption for performing a NMA is the consistency that the agreement between the direct and indirect sources of the network. We will evaluate the presence of local inconsistency and global inconsistency in ADDIS V.1.16.3. and will be duplicated in Stata V.14.0.

### 2.8 Measures for publication bias

To minimize the potential impact due to publication bias or other reporting biases, a comprehensive retrieval will be conducted and eligible studies will be chosen totally by strict standard. We will use the comparison-adjusted funnel plot to assess risk of publication bias and explore the possibility of small study effects.^[35,36]^

### 2.9 Subgroup analyses and sensitivity analyses

Where possible, we will conduct the network meta-regression meta-analyses of data on primary outcomes for the:

1. sex ratio;
2. the types of stroke;
3. the treatment duration;
4. the severity of depressive symptoms at baseline.

We will explore potential explanations in extra subgroup analyses according to the results of heterogeneity and inconsistency. Any statistical heterogeneity, when interpreting the results, will be taken into account especially if there is any variation in the direction of effect. In the sensitivity analysis, both trials where missing data have been imputed and trials where high risk of bias rating have been assessed will be excluded. And, we will not only test whether the results change but also if transitivity (consistency/model fit) is affected.

## 3 Discussion

We will assess the quality of evidence with the GRADE framework: risk of bias, heterogeneity or inconsistency, imprecision, indirectness, and publication bias.^[37]^ Two independent review authors (HWL and ZWN) will judge the quality of evidence contributing to primary outcomes (high, moderate, low or very low), while disagreements will be resolved by discussion with a third member (LXQ). Our study will generate evidence for TCM in the treatment of PSD and help to reduce the uncertainty about the effectiveness of PSD management. The results will encourage further suggestions for TCM clinical practice or guideline, which will draw wide attention.

## Author contributions

HWL and LXQ conceived the study and drafted the protocol. HWL wrote the first draft of the manuscript. LXQ, WJ, TJH assisted in protocol design and revision. HWL and SYW participated in the search strategy development. HWL, LXQ and ZWN participated in the design of data synthesis and analysis. All the authors have approved the publication of the protocol.

**Conceptualization:** Wanlin Huang.

**Data curation:** Wanlin Huang.

**Formal analysis:** Wanlin Huang, Yawei Shan, and Weini Zhou.

**Methodology:** Wanlin Huang, Xiaoqin Liao, Jinhui Tian, Jing Wu, and Yawei Shan.

**Project administration:** Xiaoqin Liao.

**Software:** Wanlin Huang, Jinhui Tian, and Jing Wu.

**Supervision:** Xiaoqin Liao.

**Writing – original draft:** Wanlin Huang.

**Writing – review & editing:** Xiaoqin Liao and Jinhui Tian.

## Supplementary Material

Supplemental Digital Content
