# Supplementary material for: Traditional Chinese medicine for post-stroke depression: A systematic review and network meta-analysis (Protocol)
Source: Medicine (Baltimore). 2018 Dec 28;97(52):e13840. doi: 10.1097/MD.0000000000013840 (PMC6314659; doi:10.1097/MD.0000000000013840)
Supplement: Supplemental Digital Content [file medi-97-e13840-s001.docx]

Traditional Chinese Medicine for post-stroke depression: a systematic review and network meta-analysis(Protocol)

Wanlin Huang^a^(Master student), Xiaoqin Liao^a^(PhD. student), Jinhui Tian^b^(PhD), Jing Wu^a^(PhD), Yawei Shan^a^(Master), Weini Zhou^a^(Master student)

Appendix 1

#1. cerebrovascular disorders/ or exp basal ganglia cerebrovascular disease/ or exp brain ischemia/ or exp carotid artery diseases/ or exp intracranial arterial diseases/ or exp intracranial arteriovenous malformations/ or exp “intracranial embolism and thrombosis”/ or exp intracranial hemorrhages/ or stroke/ or exp brain infarction/ or vasospasm, intracranial/ or vertebral artery dissection/ or “stroke”[Mesh]

#2. (stroke or poststroke or post-stroke or cerebrovasc* or brain vasc* or cerebral vasc* or cva* or apoplex* or SAH).tw.

#3. ((brain* or cerebr* or cerebell* or intracran* or intracerebral) and (isch?emi* or infarct* or thrombo* or emboli* or occlus*)).tw.

#4. ((brain* or cerebr* or cerebell* or intracerebral or intracranial or subarachnoid) and (haemorrhage* or hemorrhage* or haematoma* or hematoma* or bleed*)).tw.

#5. hemiplegia/ or exp paresis/

#6. (hemipleg* or hemipar* or paresis or paretic).tw.

#7. #1 or #2 or #3 or #4 or #5 or #6

#8. (depress* OR “affective illness” OR “mood disorder*” OR “psychiatric symptom*” OR “psychiatric disorder*” OR “affective disorder*” OR “emotional disorder*” OR dysthymi* OR “mood change*” OR “emotional disturbance*” OR “adjustment disorder*” OR “depression”[Mesh] OR “depressive disorder[Mesh]”)

#9. (poststroke depression OR post stroke depression OR depression after stroke OR PSD )[tiab]

#10. #7 OR #8 OR #9

#11. (Scraping or cupping or moxibustion with seed-sized moxa cone or sandwiched moxibustion or indirect moxibustion or suspended moxibustion or kerotherapy or keritherapy or wax therapy or point application or acupoint sticking therapy or plaster on specific points or acupoint application or meridian points therapy or traditional Chinese medicine soaking or traditional Chinese drug soaking or herb soaking or cold compress with Chinese herbs or cold compress with Chinese medicine or hot compression with traditional Chinese medicine or Chinese herb wet compressing or Chinese traditional medicine wet compress or conclusionwet application of Chinese drug or Chinese medicine fumigation or herbal fumigation or Chinese medicine iontophoresis or herb iontophoresis or Chinese traditional medicine iontophoresis or iontophoresis with traditional Chinese medicine or iontophoresis of Chinese Medicine or acupoint injection or point injection or hydro-acupuncture or aqua acupuncture or fluid acupuncture or point medicine injection or auricular point sticking or acupoint massage or traditional Chinese medicine enema or traditional Chinese medicine sausage or herb enema or herb sausage)[tiab]

#12. #10 AND #11

#13. (randomized controlled trial [pt] OR controlled clinical trial [pt] OR randomized [tiab] OR placebo [tiab] OR clinical trials as topic [mesh: noexp] OR randomly [tiab] OR trial [ti]) NOT (animals [mh] NOT humans [mh])

#14. #12 AND #13
